# Experiences of Sensory Overload and Communication Barriers by Autistic Adults in Health Care Settings

**DOI:** 10.1089/aut.2020.0074

**Published:** 2022-03-09

**Authors:** Maria Strömberg, Lina Liman, Peter Bang, Kajsa Igelström

**Affiliations:** Division of Neurobiology, Department of Biomedical and Clinical Sciences, Linköping University, University Hospital Campus, Linköping, Sweden.

**Keywords:** sensory overload, reverse empathy, health care accessibility, compensation, masking

## Abstract

**Background:**

Autistic adults have an elevated risk of many health problems compared with the general population, making health care access extra critical. Unfortunately, autistic people often find health care settings quite aversive, and many medical providers report feeling unsure about how to interact with autistic patients. We aimed at characterizing specific challenges regarding sensory experiences and communicative barriers in health care settings.

**Methods:**

We recruited adults to complete an anonymous online questionnaire on the topic of improving health care experiences for everyone. The questions covered demographics, sensory experiences in medical settings, and communication with health care providers. We quantified the associations between autism diagnosis and experiences of sensory discomfort and communication barriers in health care settings. We also did a qualitative analysis of text responses to questions on how to improve sensory environments and communication with providers.

**Results:**

Swedish adults (62 autistic and 36 nonautistic) participated in the study. The cohort was well educated, and autistic participants received their autism diagnosis late in life (median age 36 years, range 13–57). Compared with nonautistic participants, autistic participants reported greater discomfort with background sound levels in health care settings and felt more misunderstood by health care providers. Thematic analyses showed that auditory stimuli and proximity to other people were particularly bothersome for autistic participants, causing stress or avoidance and affecting the ability to interact with providers. Providers contributed to communication barriers by failing to recognize the need for individualized information, especially when respondents' difficulties were not visible or taken seriously. Participants requested greater clarity and supplementary written information. Providers also misunderstood autistic adults' body language or eye contact patterns, as they interpreted their clients through the lens of neurotypical expectations.

**Conclusions:**

Our results extend previous research by emphasizing sensory aspects of health care settings and suggesting specific and reasonable adaptations. The results also highlight how the provider's implicit expectations of nonverbal communication caused misinterpretations of autistic people who were socially skilled but did not use typical body language. Based on the data, we suggest specific adaptations, many of which may also benefit nonautistic people.

## Community brief

**Why was this study done?:**

Health care needs of autistic adults are often unmet. This may contribute to poorer health outcomes in autistic compared with nonautistic adults. Autistic differences may not be obvious in this group because of behavioral compensation strategies. Health care providers may underestimate the support needs of autistic adults, leading to decreased quality of care. By analyzing autistic adults' own experiences, we may better understand barriers to effective health care.

**What was the purpose of this study?:**

We aimed at identifying patterns of sensory and communicative experiences that autistic adults find problematic in health care settings.

**What did the researchers do?:**

In an online questionnaire, we asked autistic and nonautistic adults how they experienced various medical settings. We focused on specific sensory inputs, such as light levels and background sounds, in waiting rooms and other medical contexts. We also asked questions about communication between patients and providers. Finally, we did a qualitative analysis on free-text responses about sensory environments for both groups, and about communication for the autistic group.

**What were the results of the study?:**

Ninety-eight people (62 autistic) participated. Most of the cohort was female or gender-diverse, middle-age, and well educated. Autistic participants identified auditory inputs as one of the greatest stressors in medical settings. They discussed the impact of light levels and other people's presence on their energy levels and ability to communicate. Health care providers often misunderstood their autistic patients, leading to a failure in transferring medical information. Participants described how providers underestimated their needs, even when they were aware of the autism diagnosis. Participants wanted to get information delivered at a slower pace and with a greater amount of detail, to be better able to process medical or procedural information.

**What do these findings add to what was already known?:**

The study contributes with information on specific sensory challenges and suggests that auditory noise is particularly problematic for autistic people. On the topic of communication, the findings point to a “double empathy” problem, whereby the provider's own limitations contribute significantly to communication barriers. This was apparent in accounts of nonverbal communication, where the provider's expectations of neurotypical body language caused misunderstandings that were difficult to overcome.

**What are the potential weaknesses in the study?:**

The sample was small and comprised an ethnically narrow demographic group. Thus, the results are not generalizable to other autistic populations, such as minimally verbal adults. We also did not measure health status beyond diagnosed conditions.

**How will these findings help autistic adults now or in the future?:**

The consequences of sensory and communicative barriers may go entirely unnoticed when autistic differences are not visible. Unsuccessful interactions with the health care system may have enormous effects on the health and quality of life of autistic people. Therefore, educators and providers may use the insightful information provided by autistic participants in this study to inform decisions on staff training or design of sensory environments.

## Introduction

Autistic adults have a high rate of mental health conditions and increased mortality from suicide compared with nonautistic individuals.^1–4^ Cognitive compensation is common, masking autistic challenges and often delaying diagnosis, but autism-related challenges can severely decrease quality of life and health.^5^ Transition into adulthood appears to be a phase in which autistic people have a need for health care services, but begin to use fewer of them.^5–9^ Autistic adults are at a greater risk for a range of health concerns compared with the general population, and they utilize more health services on average.^4,10,11^ However, health care needs often go unmet.^12,13^

Nicolaidis et al. showed that autistic adults were less satisfied with patient–provider interactions and had more unmet health care needs than nonautistic adults.^13,14^ An interview study in a diverse group of autistic people and their supporters found that health care satisfaction was affected by factors on patient-, provider-, and system-levels.^7^ Patient-level factors included autism-related differences, such as communication styles and sensory sensitivities. Provider-level factors included ignorance about adult autism as well as unwillingness or inability to provide accommodations. System-level factors involved the influence of contextual and structural complexity of health care systems, as well as accessibility issues and societal stigma.^7^

We aimed at contributing to this topic by focusing on patient- and provider-level factors in autistic adults who were able to participate in an online study. Autistic challenges in this population may be difficult to recognize, but sensory and communicative differences could critically affect health care satisfaction and accessibility. We asked Swedish adults about their perception of health care settings, focusing on sensory inputs and patient–provider communication. We hypothesized that autistic adults would report greater levels of sensory adversity and communication difficulties in health care settings compared with a nonautistic group. Further, we used qualitative analysis of free-text responses with the aim of better understanding the nature of sensory and communication challenges.

## Methods

### Participants

We recruited participants by using social media, inviting “women, men and transgender people, with and without neurodevelopmental diagnoses.” Exclusion criteria were self-reported neurodegenerative disorders and psychotic illness, but no one reported these conditions. We excluded participants who provided incomplete or inconsistent information, or who did not know what diagnoses they had (28 participants, see Supplementary Data for details). For the current analysis, we also excluded individuals without a formal diagnosis who self-identified as autistic.

The advertised aim of the study was to “help improve environments, communication and knowledge in health care situations.” We intentionally phrased the aims and study materials without any reference to autism, to decrease recruitment biases. The inclusion of nonautistic people provided information on whether findings were specific to autistic people. We describe the recruitment strategy and quality control in detail in the Supplementary Data. According to Swedish regulations, the study was exempt from ethical review due to the anonymity of participants. Thus, an ethical board did not evaluate the study. We designed the questionnaire in accordance with the Declaration of Helsinki.

### Measures

The content and organization of the questionnaire are described in detail in the Supplementary Data.

#### Background questionnaire

We obtained information on age, gender, education, work status, and family/living conditions. We also asked follow-up questions with the purpose of identifying inattentive responders. We used the number of self-reported psychiatric and somatic conditions as a proxy of individual health burden. To obtain further descriptive data on the cohort, we used the 10-item Autism Quotient (AQ)^15^ to obtain a dimensional measure of autistic characteristics. The main purpose of including the AQ was to support quality control; major deviations from previously published distributions in autistic and nonautistic populations would indicate sampling bias or other problems with recruitment. A 4-point scoring system gave a dimensional AQ score between 10 and 40 (AQ10-dim).

#### Sections on health care experiences

Our neurodiverse team identified relevant health care-related questions, based on experience with outreach and educational work. An autistic team member carefully refined the questionnaire to minimize ambiguity and open-endedness. To make the questionnaire logical to follow, we organized questions into three sections: sensory environments, communication with providers, and providers' knowledge about autism.

Even though our primary goal was to analyze free-text responses, we knew from previous questionnaires that many autistic participants struggle with textboxes, due to their open-endedness. Therefore, each section began with a few multiple-choice questions (listed in Supplementary Table S1) to help participants ease into the topic and allow us to collect data from participants who were unwilling to write free text. To decrease open-endedness, we constructed statements to put sensory and communicative experiences into specific environments. For example, we presented the statement “I have usually found the light levels acceptable” several times for different contexts (e.g., “In waiting rooms in psychiatric outpatient clinics…”). Environments included waiting rooms, examination rooms, common rooms, and patient rooms. Settings included outpatient/inpatient and somatic/psychiatric health care clinics. Similarly, we repeated communication statements (e.g., “Medical staff actively try not to deviate from what I was prepared for”) for the different settings, without specifying room types. Response options were Disagree, Somewhat Disagree, Neutral, Somewhat Agree, and Agree (scored 1–5).

We repeated the open-ended question “Based on your experiences, how could the environment in [room type] be improved?” once for each type of room (waiting rooms, examination rooms, common rooms, and patient rooms). We asked the following two questions on communication to both nonautistic and autistic participants:

- “How would you like to be communicated with/treated in healthcare situations?”
- “How could communication in healthcare situations be improved?”

We asked autistic participants only about their health care providers' autism-related attitudes, knowledge, and skills. The questions were:

- “Have you experienced that autistic difficulties, behaviors or special needs have been handled in an inappropriate manner by healthcare providers? If so, please describe how.”
- “Has your autism been questioned by healthcare providers, even when they knew you have a diagnosis?” (Yes/No, with textbox to specify).
- “What knowledge about autism do you think that healthcare providers should have?”
- “Have you experienced a lack of knowledge about autism in [psychiatric/somatic] healthcare settings? If so, please give examples.”

None of the questions were mandatory.

### Procedures

Potential participants followed a link to an information page, which also contained a link to a questionnaire containing the background questions. At the end of these sections, participants received a randomized subject ID and continued to a landing page on our website. This allowed participants to take a break before clicking on a link to a questionnaire that contained the health care questions. Here, they identified themselves by pasting their subject ID into a response box. The detailed structure and content of the questionnaires are available in the Supplementary Data.

### Data analysis

We used GraphPad Prism 8 (GraphPad Software, San Diego, CA) and JASP (JASP v.0.14.1; JASP Team, University of Amsterdam, NL) to analyze data. We compared age and AQ between groups by using unpaired *t*-tests, and health burden (No. of conditions) by using a Mann–Whitney test. The Cronbach's *α* was 0.851 for the AQ, indicating good data quality.

We did not compare different rooms or settings, because unmeasured factors would confound this. Instead, for each participant, we pooled responses to each sensory or communication statement (e.g., “I have usually found the light levels acceptable”) into a binary variable that showed whether the participant disagreed (score 1–2) with the statement in one or more contexts. We used these binary variables as dependent variables in multivariable logistic regressions to examine whether they were associated with the presence of an autism diagnosis. A first model contained only autism as an independent variable to derive odds ratios (ORs) relative to the absence of autism, and a second model contained both autism and age. Because attention deficit/hyperactivity disorder (ADHD) is often associated with autism as well as atypical sensory processing, we added ADHD to a third model. In a final model, we added anxiety and depressive disorders to adjust for possible associations between affective conditions with sensory and communicative experiences.

We performed a semantic-level, reflexive thematic analysis on free-text responses, using a postpositivist paradigm.^16,17^ We used Quirkos software (Quirkos Ltd., Edinburgh, Scotland) and NVivo 12 (QSR International, MA). To enhance credibility, two investigators (M.S., K.I.) familiarized themselves independently with all data without access to demographic or clinical information. We generated preliminary codes individually before the first group discussion, giving equal attention to all the text. We refined the codes through discussions and used them to recode the material. We repeated this process several times and organized peer debriefing meetings to identify biases in our understanding of the material. When we had coded all material, we identified broader themes in several group discussions. The significance of themes was determined subjectively by our research group, requiring that (1) multiple autistic participants expressed it as a salient issue, and (2) it was related to experiences of sensory inputs or interactions with providers. To address possible bias introduced by our team's prior knowledge of autism-related challenges, we also involved several external collaborators with contrasting professional and personal backgrounds in discussions about how the raw material related to codes and themes.

In developing themes, we remained blinded to whether participants had an autism diagnosis until after agreeing on themes. This helped ameliorate confirmation bias in our coding. Once we had decided on themes, we used Qualtrics query functions to print the material pertaining to each group and evaluated whether themes were present across groups or only in one. We report this group comparison for the sensory items, as both groups responded meaningfully to them. For the communication items presented to both groups, nonautistic participants' contribution was too sparse for a meaningful comparison. In a final step, we recoded the entire corpus of data to capture all information pertaining to the themes.

## Results

### Participants

Out of the 98 eligible participants, 62 reported a diagnosis of autism. Most participants were born in Sweden, cisgender female and had graduated from at least high school (Table 1). The average age was lower in the autistic group, but other demographic variables were not significantly different (Table 1). The most common co-occurring conditions were anxiety disorders, ADHD, depressive disorders, and exhaustion disorder (code F43.8 in the Swedish version of ICD-10, “other reactions to severe stress”^18^) (Table 2). The AQ10-dim score was significantly higher in the autism group than in the nonautistic group [mean ± SE: 28.3 ± 3.9 vs. 21.1 ± 3.5, *t*(96) = 9.091, *p* < 0.0001]. The health burden was higher in the autistic group (median 3 vs. 1 co-occurring conditions, Mann–Whitney test, *p* = 0.0005). In the autism group, the median number of years since diagnosis was 4 (range 0 to 20), corresponding to a median age at diagnosis of 36 years (range 13–57).

**Table 1.** Descriptive Demographic Data

	All participants	Nonautistic	Autistic	Test statistic
*N*	98	36	62	
Age (mean ± SD)	44.0 ± 11	49.5 ± 11	40.8 ± 11	*t*(96) = 3.89, ***p* < 0.01**
Gender (cis-female, cis-male, gender-diverse)	72, 9.2, 18	83, 8.3, 8.3	66, 9.7, 24	*χ*^2^(2) = 4.09, *p* = 0.13
Born in Sweden (%)	92	89	94	*χ*^2^(1) = 0.66, *p* = 0.42
Finished high school (%)	93	97	90	*χ*^2^(1) = 1.64, *p* = 0.20
Full-time work or studies (%)	38	56	27	***χ***^2^(1) = 7.67, ***p* < 0.01**
Full-time disability/sick leave (%)	31	17	39	***χ***^2^(1) = 5.21, ***p* = 0.02**
Married or domestic partner (%)	55	61	52	*χ*^2^(1) = 0.69, *p* = 0.41
Children <18 years living at home (%)	46	56	40	*χ*^2^(1) = 2.13, *p* = 0.15

Bold values indicate the statistical significance at *p* < 0.05.

**Table 2.** Frequency of Co-occurring Conditions

	All participants (%)	Nonautistic (%)	Autistic (%)	Test statistic
Anxiety disorder	43	17	58	*χ*^2^(1) = 15.9, ***p* < 0.01**
ADHD	35	19	44	**χ**^2^(1) = 5.84, ***p* = 0.02**
Depressive disorder	33	14	44	**χ**^2^(1) = 9.11, ***p* < 0.01**
Exhaustion disorder (F43.8A, ICD-10)	33	22	39	*χ*^2^(1) = 2.82, *p* = 0.09
One or more somatic conditions	32	31	32	*χ*^2^(1) = 0.03, *p* = 0.86
Post-traumatic stress syndrome	17	17	18	*χ*^2^(1) = 0.02, *p* = 0.89
Eating disorder	7.1	2.8	9.7	*χ*^2^(1) = 1.64, *p* = 0.20
Dyslexia	5.1	2.8	6.5	*χ*^2^(1) = 0.64, *p* = 0.43
Obsessive/compulsive disorder	4.1	0	6.5	*χ*^2^(1) = 2.42, *p* = 0.12
Personality disorder	4.1	2.8	4.8	*χ*^2^(1) = 0.25, *p* = 0.62
Bipolar disorder	3.1	2.8	3.2	*χ*^2^(1) = 0.02, *p* = 0.90
Tourette's syndrome/tic disorder	2.0	0	3.2	*χ*^2^(1) = 1.19, *p* = 0.28
Developmental coordination disorder	1.0	0	1.6	*χ*^2^(1) = 0.59, *p* = 0.44
Intellectual disability	1.0	0	1.6	*χ*^2^(1) = 0.59, *p* = 0.44

Bold values indicate the statistical significance at *p* < 0.05.

ADHD, attention deficit/hyperactivity disorder.

### Association between autism and dissatisfaction with sensory and communicative factors in health care settings

Autistic participants were more likely than nonautistic participants to find background sound levels unacceptable, and this difference remained significant after accounting for the presence of ADHD and anxiety/depression (Table 3). Autistic participants also commonly reported intolerable light levels, but this was better accounted for by ADHD (OR 3.30, confidence interval [CI] 1.11–9.79, *p* = 0.032). Autism was associated with being disturbed by sensory impressions from other people and not feeling safe in health care settings, but the differences were not significant after including other variables in the model (Table 3). Autistic participants were more likely to feel misunderstood in health care settings, have trouble finding directions in outpatient clinics, and be unsure about how to get medical attention in an emergency (Table 4). However, the differences did not remain significant in the regression model that included co-occurring conditions. Similarly, autistic participants did not think that providers proactively helped avoid unexpected changes, but the ORs were not significant after taking other variables into account (Table 4).

**Table 3.** Odds Ratios (and 95% Confidence Intervals) for the Association of Autism with Measures of Sensory Discomfort in Health Care Settings

Dependent variables	Model 1: Autism (unadjusted)	Model 2: Autism (adjusted for age)	Model 2: Autism (adjusted for age+ADHD)	Model 3: Autism (adjusted for age+ADHD+anxiety+depression)
Disagreement with “I have usually found the light levels acceptable.”	**2.96 (1.25–6.99)** ***p* = 0.013**	**2.81 (1.12–7.08)** ***p* = 0.028**	2.41 (0.92–6.27) *p* = 0.073	2.37 (0.86–6.54) *p* = 0.097
Disagreement with “I have usually found the background sounds acceptable.”	**3.71 (1.56–8.81)** ***p* = 0.003**	**2.87 (1.15–7.23)** ***p* = 0.024**	**2.76 (1.09–7.02)** ***p* = 0.033**	**3.49 (1.25–9.72)** ***p* = 0.017**
Disagreement with “I have rarely been bothered by visual impressions (e.g., colors, art, decorations).”	2.07 (0.90–4.77) *p* = 0.087	1.84 (0.75–4.48) *p* = 0.183	1.61 (0.65–4.03) *p* = 0.307	1.37 (0.52–3.64) *p* = 0.525
Disagreement with “I have rarely been bothered by sensory impressions from other people.”	**2.33 (1.00–5.39)** ***p* = 0.049**	2.06 (0.84–5.06) *p* = 0.114	1.99 (0.80–4.96) *p* = 0.139	1.89 (0.73–4.93) *p* = 0.191
Disagreement with “The volume of the healthcare provider's voice has usually been at a moderate level.”	2.61 (0.98–6.90) *p* = 0.054	2.32 (0.83–6.44) *p* = 0.107	2.19 (0.78–6.15) *p* = 0.139	1.75 (0.59–5.20) *p* = 0.313
Disagreement with “I have usually felt safe.”	**2.84 (1.20–6.72)** ***p* = 0.018**	2.30 (0.93–5.72) *p* = 0.073	2.07 (0.82–5.25) *p* = 0.125	1.89 (0.71–5.00) *p* = 0.202

Each row represents logistic regression results for one binary dependent variable. Each column represents one regression model with a specified set of independent variables. We progressively added collinear and potentially confounding variables. ORs and CIs above 1 indicate a significantly higher disagreement in autistic people. Statistical significance is emphasized by bold text.

CIs, confidence intervals; ORs, odds ratio.

**Table 4.** Odds Ratios (and 95% Confidence Intervals) for the Association of Autism with Measures of Communicative Challenges in Health Care Settings

Dependent variables	Model 1: Autism (unadjusted)	Model 2: Autism (adjusted for age)	Model 2: Autism (adjusted for age+ADHD)	Model 3: Autism (adjusted for age+ADHD+anxiety+depression)
Disagreement with “*I rarely feel misunderstood within [psychiatric/somatic] [inpatient/outpatient] settings*.”	**3.26 (1.29–8.23)** ***p* = 0.012**	2.08 (0.77–5.65) *p* = 0.151	2.00 (0.73–5.50) *p* = 0.181	2.75 (0.88–8.57) *p* = 0.081
Disagreement with “*If I need acute [psychiatric/somatic] care, I know how it works and what to expect.*”	**3.33 (1.39–8.00)** ***p* = 0.007**	**2.73 (1.09–6.84)** ***p* = 0.032**	**2.66 (1.05–6.75)** ***p* = 0.040**	2.27 (0.86–6.00) *p* = 0.100
Disagreement with “*When I arrive at a [psychiatric/somatic] outpatient clinic, it is clear how I get to the right place*.”	**2.91 (1.20–7.11)** ***p* = 0.019**	**2.67 (1.04–6.81)** ***p* = 0.041**	2.35 (0.90–6.14) *p* = 0.080	2.17 (0.77–6.12) *p* = 0.142
Disagreement with “*Healthcare providers in [psychiatric/somatic] [inpatient/outpatient] settings actively try to minimize deviations from what I was prepared for.”*	**3.34 (1.33–8.42)** ***p* = 0.011**	**2.77 (1.05–7.35)** ***p* = 0.040**	2.62 (0.98–7.02) *p* = 0.055	2.47 (0.88–6.95) *p* = 0.087
Disagreement with “*Healthcare providers in [psychiatric/somatic] [inpatient/outpatient] settings are usually understanding and helpful if I have trouble with deviations from what I was prepared for.*”	2.43 (0.94–6.30) *p* = 0.069	2.16 (0.79–5.92) *p* = 0.135	2.03 (0.73–5.62) *p* = 0.175	1.55 (0.53–4.54) *p* = 0.427

Each row represents logistic regression results for one binary dependent variable. Each column represents one regression model with a specified set of independent variables. We progressively added collinear and potentially confounding variables. ORs and CIs above 1 indicate a significantly higher disagreement in autistic people. Statistical significance is emphasized by bold text.

### Thematic analysis of text responses

Fifty-eight participants (44 autistic, 14 nonautistic) provided free-text responses to sensory questions, whereas 55 autistic participants responded to questions about communication and knowledge. We present the themes next.

#### Sensory processing theme: reducing input intensity and clutter

This theme encapsulated descriptions of how sensory load affected health care experiences, as well as suggestions for how to minimize negative effects. Autistic participants addressed the topic more extensively than nonautistic participants, and there were qualitative group differences.

The most noticeable difference was in the auditory domain, which nonautistic participants rarely addressed at all. Autistic participants provided extensive details on the type of difficulties they experienced. Overall, they perceived sound levels as stressful and exhausting. Common specific sources of stress and overload were TVs, alarms, phones, and ticking clocks. There were many comments about needing sound-insulation between rooms and sound-absorbing materials inside rooms. Suggestions included using headphones for the TV/radio, using quieter phones and ventilation, and taking steps to prevent echoes.

Participants in both groups mentioned that they would like less sharp light. Qualitatively, autistic participants provided more details, including adversity to bright or flickering lights, such as fluorescent/sharp lamps or decorative Christmas lights. Suggestions included softer global lighting, small lamps, and fewer reflective surfaces. Participants perceived environments as especially stressful when light levels were out of the individual's control, such as in waiting rooms and shared patient rooms. Autistic participants appreciated when providers proactively offered to turn down the lights, in part because they found it difficult or embarrassing to ask.

*In the psychiatric clinic, they know that I don't like fluorescent lights and have sometimes turned off the ceiling light when I come in. I appreciate that, it makes it easier for me to focus on the conversation. (Autistic gender divergent, 35)*

Both groups disliked clutter and unpleasant colors, but autistic participants focused more on how this affected their socio-cognitive abilities. Irrelevant stimuli such as artwork, equipment, or events outside the window pulled attention away and made communication difficult.

Participants discussed tactile and olfactory modalities less often than visual and auditory inputs. Olfactory input was a source of emotional adversity or sensory overstimulation in the autistic group. Participants in both groups mentioned temperature and ventilation as important environmental factors, and some autistic participants found the tactile feeling of blankets in patient rooms bothersome.

#### Sensory processing theme: maintaining calm and predictability in personal space

The first theme was related to the intensity of stimuli, whereas the second theme encapsulated effects of sensory unpredictability related to human factors. Nonautistic participants contributed in a minor manner to this theme in our material. Odors, movement, and other inputs from people nearby generated a sense of unpredictability and stress in autistic participants. Some wanted to wait in a separate room or outside the clinic. Many suggested more subtle improvements such as using armchairs instead of couches, dividing rooms into smaller sections, and ensuring seats did not face each other. Shared inpatient rooms were stressful, because they removed the ability to control the environment.

*There should always be chairs/spots where no one can sit down next to you, so that you can sit alone. There should be possibilities to have a wall behind your back so that people can't sit or be behind you. (Autistic female, 45)*

#### Communication theme: we speak different languages—please meet me halfway

This theme encompassed concepts related to information transfer in patient–provider interactions. Participants expressed that they needed individualized information to help them prepare for health care situations, such as a letter ahead of time that detailed the procedures and showed photos of facilities and staff. Other helpful advance information included the duration of the visit, the structure of the conversation, and explanations of medical procedures. Literal thinking and the inability to “read between the lines” contributed to communication problems, and participants wished that providers would help bridge the gap by increasing the level of detail in their communication. Providers instead routinely underestimated these needs.

*It would be nice to get exceedingly clear instructions. […]. ‘Have a seat’ vs ‘sit down there and wait’ are very different. Especially if there are different places to sit […]. Also, knowing if one should pay immediately or not until afterwards or what's about to happen. (Autistic female, 55).*

Another issue was that of nonverbal communication. Autistic individuals expressed that there existed an implicit assumption that their body language and tone of voice added important social information. Some autistic individuals expressed that their body language or facial expressions did not match the meaning of their words, and they felt misunderstood when the doctor unconsciously put too much weight on projected nonverbal communication. Providers could also hear messages “between the lines” even when there were none. Individuals wished that providers would be able to put aside their habit of making interpretations of nonverbal information, and instead focus on what their patient literally said.

*Listen to what I say, even if my facial expressions don't always match my words (Autistic gender divergent, 21)*

Patient–provider conversations were prone to misunderstandings. Failure to convey and receive information introduced errors in medical records. Participants appreciated when the provider actively confirmed that they had understood the information correctly. Several participants expressed a reluctance or fear of asking additional questions.

*I would like to get the question how I've experienced the visit, so that they know whether or not I have taken in all the information and whether or not I have been able to express myself fully […] (Autistic male, 48)*

Participants suggested alternative means of communicating. Written information about past, present, and future was very helpful, such as written information in advance and getting a written summary from the visit. Several participants requested visual aids, such as drawing on a whiteboard or using pictures to communicate, or permission to make audio recordings.

*When I have been admitted to a ward, I would have liked to have their schedule written down. What time is breakfast/lunch/food? When do they make rounds? (Autistic female, 41)*

Some participants also mentioned wanting higher-level individualized adaptations. For example, one participant wanted providers to adapt to his preference for using computer analogies to communicate about himself. Another person wanted to receive psychiatric care online in written form, as she avoided much-needed treatment due to the stressful environment at medical centers.

Finally, the communication barriers described earlier amplified when a treatment involved multiple providers, due to the need to “start over” with every new person. Such lack of continuity may be detrimental for any patient, but autistic people may be especially vulnerable.

#### Communication theme: the need to feel safe and stress-free

This theme encapsulated interactions between providers' personal qualities and the needs of the patient. Incompatibilities triggered emotional reactions.

Contributing to high stress levels for autistic participants was an inability to cope with the high pace of communication, examination or decision making, and a general inability of providers to accommodate for slow processing. The provider had an important role in managing or amplifying this stress. To reduce stress and improve communication, the participants suggested that they would benefit from additional time in appointments and conversations. In addition, general stimulus reduction was helpful to decrease stress and allow for undisturbed information processing.

*Filling out forms (*e.g., *in the waiting room) to get as much information as possible. It gives better responses because you get to think it through. Questions that I never thought about before have usually [resulted in] the wrong answer, because I'm too stressed to give an answer fast. (Autistic female, 53)*

Participants perceived preexisting psychiatric diagnoses, including autism, as a barrier to effective interaction and treatment. In part, they attributed this to a tendency for some providers to get distracted from the cause of concern when a coexisting psychiatric challenge was present, sometimes to the point of labeling a condition as psychosomatic without an examination. This caused fear or anger, negatively impacting communication. Autistic participants also expressed that providers questioned their autism diagnosis based on superficially high levels of functioning. Regardless of the providers' intentions, participants perceived this type of input as deeply unsettling and a cause of insecurity.

*[…]That they don't question physical symptoms just because you have a psychiatric diagnosis. That you don't have to defend things that are part of the [autism] diagnosis. […] (Autistic female, 40)*

*I want them to ASSUME that what I tell them about myself is true, honest and reasonable. They often don't. And that they accept a seriously given [autism] diagnosis. (Autistic male, 50)*

Commonly, interpersonal factors, such as the provider's attitude and ability to listen, dominated the overall experience. Participants expressed how negative interpersonal experiences made them less trusting and more anxious about their ability to get the care they needed.

#### Knowledge theme: increasing awareness of heterogeneity and camouflaging in autistic adults

Forty percent of the autistic participants responded “Yes” to the direct question about whether medical professionals had questioned their autism despite a documented diagnosis. Participants reported that health care providers often lacked knowledge about autism, and this affected their health care negatively. Health care providers were not always able to combine their professional knowledge of the autism spectrum with the social impression of a verbal, well-educated patient. This affected the autistic adults negatively as they felt unseen, disrespected, or questioned. Health care providers generally did not arrange for adaptations spontaneously.

Participants listed several classical autistic challenges as factors that providers should be aware of, such as difficulties with unpredictability or social jargon. Common challenges were unplanned delays, open-ended questions, hypersensitivity to medicines, and atypical perception or expression of pain. Many participants indicated that they would be satisfied with a relatively basic understanding of autism, and that it was more important that the provider understood the heterogeneity of the spectrum. Knowledge about individual variability was severely lacking, causing stigma and miscommunication. Several individuals also pointed out that the fact that their autism had been discovered late in life probably indicated a gap between existing and desired knowledge.

## Discussion

Our study characterized specific sensory challenges and communicative barriers experienced by autistic adults in health care situation. Compared with the nonautistic control group, autistic participants were more likely to find sensory, particularly auditory, stimuli aversive in health care settings. The autistic group described communicative challenges, often related to a conflict between their communication preferences and the providers' habits and expectations.

Although limited by a small sample and a limited selection of questions, our quantitative comparison between autistic and nonautistic participants highlighted auditory overload and communicative barriers as relatively specific to autistic participants. A previous study also found significant group differences on questionnaire items concerning communication and sensory challenges in health care settings.^14^ We additionally examined the influence of ADHD and anxiety/depression disorders and found possible contributions of attention deficits to sensitivity to visual inputs, whereas auditory overload appeared more specific to autism in this sample.

Sensory sensitivities may, in and of themselves, cause stress in health care settings, but they may also have cascading effects on communicative or social functions.^19^ Participants in a previous qualitative study described the direct effects of sensory sensitivity on the success of health care interactions.^7^ Our finding that autistic participants struggled, particularly with noise levels, might be related to biological differences in arousal levels or sensory habituation.^20^ Our qualitative analysis indicated that light was a source of discomfort in both groups, which may be related to known effects of indoor lighting on mood and cognition in general.^21^ However, we did not make any controlled comparison of the degree of discomfort related to varying levels of sensory inputs.

Many of the sensory difficulties described by autistic participants would be avoidable through minor environmental or procedural adaptations that would benefit most people. For example, the light environment could be modified to be more accessible and attractive to both autistic and nonautistic people (e.g., evenly distributed full-spectrum light, nonreflective surfaces), minimizing the need for individual adaptations.^22^ Visual stress could be decreased by avoiding clutter and fluorescent/flickering/sharp lamps, and by using matte paint and nonreflective surfaces. The noise environment could quite easily be improved by removing televisions and ticking clocks, and using sound-absorbing surfaces. Given our findings that auditory inputs were particularly bothersome for autistic people, such adaptations may greatly improve the health care experience. A notification system that allowed patients to wait outside the waiting room would create flexibility in the degree of exposure. The need for physical distance and sensory shielding from other patients may be related to social insecurities as well as difficulties with sensory unpredictability. Architectural approaches, such as compartmentalization and acoustical modification, were previously found to benefit cognition and mood in autistic children.^23^ In line with this, participants suggested many design solutions that would help them, such as the placement of furniture in a way that encouraged distance.

Communication difficulties described by the participants were broadly consistent with previous studies that described a lack of accessible communication and trouble with fast-paced information processing.^7^ Our participants, in addition, outlined communicative problems that were dependent on the provider's limitations in mentalizing, creating a “relational” impairment.^24^ This phenomenon has been dubbed a “double empathy problem,” as it is caused by a difference in the dispositions of both actors, and it thus affects both sides.^25^ In our data, the double-empathy problem was evident in two ways. First, providers assumed that the autistic patients used nonverbal communication in a neurotypical manner. By reading and responding to body language, facial expressions, and tone of voice, providers failed to understand that the autistic person's use of such cues may be different. This may be particularly devastating in emergency situations, where the autistic person may communicate physical pain or other needs in ways that a neurotypical doctor is unable to recognize. Second, providers tended to underestimate support needs, failing to spontaneously adjust their communication to remove metaphoric or ambiguous language. A lack of general knowledge of autism or a failure to recognize invisible autistic limitations may explain this. Given previous studies on autistic people's interactions with the health care system as well as cognitive masking of communicative deficits, a combination of these factors is likely to contribute.^7,13,26–30^

Late diagnosis and behavioral compensation for autistic traits are risk factors for poor mental health.^31,32^ Therefore, health care providers must be able to recognize and adapt to an autistic communication style when faced with a cognitively skilled autistic patient. Several studies have shown that many clinicians lacked the knowledge to interact well with autistic patients.^26,27,33^ Training in autistic communication may be useful (e.g., nonambiguous literal language, redundant multimodal information), but also a willingness to accept and adapt to diagnosed autism, including in cases where autistic traits appear to be absent. It is also conceivable that a more neurodiverse fleet of health care professionals would be better able to cater to the diverse needs of both autistic and nonautistic patients.^25^

Limitations of the study included its observational nature, the relatively small sample, and a selection bias toward a narrow demographic group. Although the inclusion of a nonautistic group provided support for autism specificity of some challenges, the comparison is preliminary as the groups were small and not matched on health status or exposure to health care settings. In addition, confounding variables were highly collinear with autism, making it possible that a lack of significance in Tables 3 and 4 reflected reduced statistical power as the number of predictors increased. The reasons for poor health in autistic populations are likely multifactorial, and we need large, well-controlled experiments to address the potential role of health care access. The sampling bias of this study caused the cohort to be dominated by motivated, highly educated females. Thus, the results do not necessarily apply to other autistic populations, such as minimally verbal people. The anonymous and text-based nature of the study prevented member checking to increase the trustworthiness of the thematic analysis. To counteract these issues, we maintained a conservative interpretation of text data. The small sample size made statistical comparisons vulnerable to both Type I and Type II errors. These difficulties were ameliorated to some extent by the narrow demographic slice, which decreased the variance within demographic dimensions (compared with a cohort representing the entire spectrum).

Representation of females, gender-diverse, and older people is rare in the autism literature, but we reached these populations effectively. Considering recent discussions about delayed diagnosis and high health burden in autistic females,^3,34^ we note that Internet-based recruitment through social media communities appears to be a valuable way to reach these underrepresented populations.

In summary, this study emphasizes that autistic people's health care experiences can be stressful and inefficient, impacting health and well-being. With relatively straightforward modifications to the light and sound environments, health care systems could provide better conditions for autistic people to communicate about their health. We suggest that it may also be beneficial to develop educational interventions for medical staff that improve providers' theory-of-mind regarding autistic cognitive styles.

## Supplementary Material

Supplemental data
