# Supplementary material for: Experiences of Sensory Overload and Communication Barriers by Autistic Adults in Health Care Settings
Source: Autism Adulthood. 2022 Mar 9;4(1):66–75. doi: 10.1089/aut.2020.0074 (PMC8992902; doi:10.1089/aut.2020.0074)
Supplement: Supplemental data [file Supp_Data.pdf]

Experiences of sensory overload and communication barriers by autistic adults in healthcare settings

Maria Strömberg[1], Lina Liman, Peter Bang[1], & Kajsa Igelström[1]*

[1] Division of Neurobiology, Department of Biomedical and Clinical Sciences, Linköping University, University Hospital Campus, 581 85 Linköping, Sweden

* Corresponding author

Supplementary Methods

Recruitment

The primary recruitment venue was the Swedish branch of the outreach project *Extraordinary Brains*. This project had been founded in the USA in early 2017 by the senior author while working at Princeton University. At its inception, the project was targeted towards neurodivergent adults, and used a combination of informal questionnaires, blog articles, and a Facebook page to establish connections with autistic communities in English-speaking countries. While the initial aim was to create a venue for study recruitment, hundreds of autistic adults (predominantly female or nonbinary) spontaneously began submitting substantial and informative feedback. We became better aware of the views around autism terminology and research priorities of autistic adults. Several autistic and transgender advisors in the UK and USA have been involved in various phases of the project. When the Swedish platform opened in 2018, it attracted a very similar demographic group to the US-based one. However, due to a wave of outreach activities and media attention, it also attracted a broader range of health and education professionals, parents and non-autistic neurodivergent people. At the time of writing, the Swedish Facebook page had 1,736 followers (even though the outreach activities have slowed down substantially due to other commitments).

We collected responses between May and September 2019, using the Qualtrics platform (Qualtrics LLC, Seattle WA, United States). The advertisement text read as follows: *"Do you want to help improve environments, communication and knowledge in healthcare situations? Do our questionnaires! We welcome women, men and transgender people, with and without neurodevelopmental diagnoses."* Study ads were shared on social media in ways beyond our control, resulting in snowball sampling. Potential participants were directed to our website, where there was a background text: "*The world can be difficult to navigate, especially for those with a neurodivergent brain or non-binary gender identity. In medical*

settings, we are especially vulnerable, making it extra important that the people around us treat us well, and that sound, light and smells have a pleasant level. Do you want to contribute to deeper insights into what does and does not work, when it comes to sensory inputs, knowledge, and communication in various medical settings? Then do our questionnaire!" The advertisement was seen on Facebook by 13,678 people and 1,448 people engaged with it by clicking or sharing it. If a prospective participant chose to view the questionnaire in Qualtrics, a more thorough introductory page was displayed. There, we explained which topics would be covered in the questionnaire and informed about ethics, anonymity, and data protection.

Macrostructure of the questionnaire

We divided the questionnaire into two submissions to allow participants to take a break in the middle. In the first half of the study, participants were given a randomly generated 16-digit subject ID, which they used to identify themselves at the beginning of the second half of the study. We used the subject ID to merge each subject's submissions before analysis.

The macrostructure of the study is shown in **Figure S1**. The first questionnaire (**Fig. S1A**) began with a section about demographic data (part 2) and two pages about participants' clinical background (part 3), followed by a quality of life (QoL) questionnaire (not included in this study) as well as the 10-item autism quotient (AQ) (parts 4-5). The second questionnaire contained healthcare questions (**Fig. S1B**). It contained three main pages, covering sensory inputs (part 4), communication (part 5), and knowledge about autism (part 6). The different parts are described in more detail below.

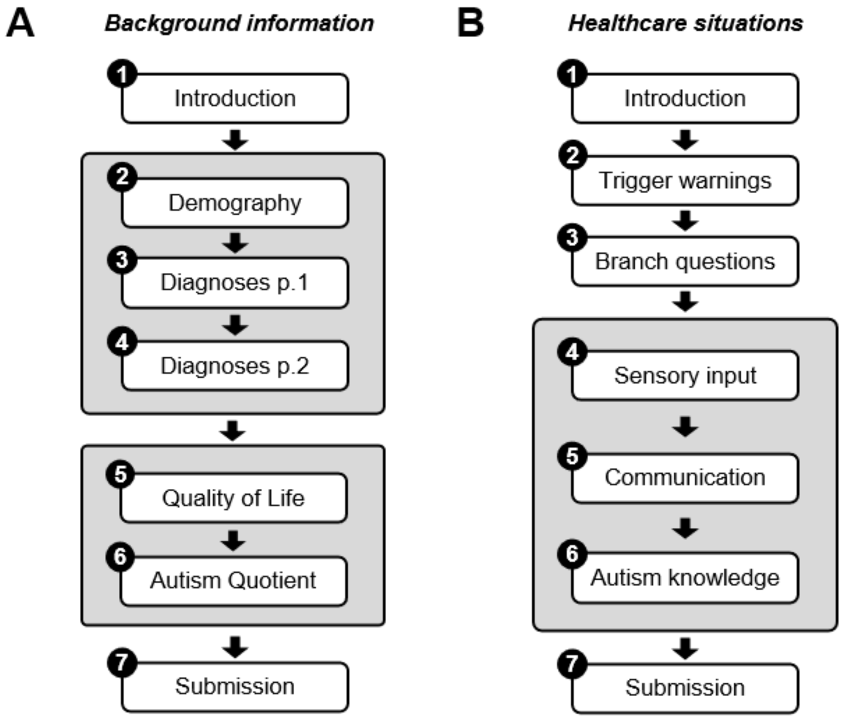

Figure S1. Overview of questionnaire flow. A. An introduction was followed by three pages of questions covering demographic questions and questions about autism and other diagnoses. After this, a QoL scale and the AQ-10 were administered. **B.** In the healthcare questionnaire, the main questions were divided into three topics: sensory inputs, communication and knowledge.

Demographic and clinical questions

In the demographics section, we asked participants about age, sex assigned at birth, gender identity, country of birth, education, professional status, marital/relationship status and number of children living at home. Gender was coded as “male”, “female” and “gender divergent”. After the demographic section, subjects were asked to choose whether they wanted to skip questions about which diagnoses they had received. The choice to omit this section was provided to decrease the risk of careless responding by unmotivated participants. This page also let participants disclose that they did not know about their diagnostic status, which allowed us to distinguish an absence of diagnoses from a lack of knowledge of them. Only subjects that marked *“Yes, I would like to see the questions about diagnoses*” were presented with the questions. Subjects that marked *“I don’t know which diagnoses I have”* or who did not consent to view the questions were excluded from the published study.

On the next page, participants marked which neurodevelopmental or psychiatric diagnoses they had been diagnosed with. For each condition listed, we provided *“Yes” and “No”* options, as well as the options *“No, but I or someone else suspects it”* and *“Yes, but I think it is incorrect”*. This format was chosen because it allowed participants to 1) disclose formal diagnoses without endorsing them, and 2) being honest about lacking a formal diagnosis. While we excluded self-identifying participants from the current publication, we viewed their input as important preliminary data.

We used display logic to administer personalized follow-up questions about the conditions that had been marked as relevant in some way (not marked as *“No”*). Follow-up questions were used mainly for preliminary analyses and are therefore not described extensively in the current study. However, the follow-up questions did provide an extra opportunity to assess data quality; we did not find any red flags in participants’ responses. Participants who had indicated that they had a formal autism diagnosis were presented with follow-up questions about the age of diagnosis, the region in which the diagnosis was

made, the exact name of the diagnosis, and the profession of the person(s) who gave them the diagnosis. Only participants who responded to all these questions were included in the autistic group.

AQ questionnaire

The 10-item AQ was used to obtain quantitative measures of autistic characteristics. Because the psychometric properties and internal consistency of these questionnaires were well known, we reasoned that the Cronbach's alpha coefficient calculated from our respondents could be used as a quality control index: The internal consistency would likely be low in the case of inattentive participants. The AQ-10 was used to look at the group-level distributions of scores, as we have previously noticed that significant quality problems (e.g., dishonest participants) tend to cause unrealistic distributions of the AQ. We also administered an 18-item QoL questionnaire to address subjective well-being. On reviewers' request, the QoL data were excluded from the present study, but successful completion of the QoL questionnaire was one of the inclusion criteria (quality check).

Trigger warning

The healthcare questionnaires contained questions about potentially sensitive topics, such as aversive events in psychiatric clinics or emergency rooms. Since the questionnaire was anonymous, we could not monitor or help participants with emotions triggered by the topics. Therefore, before presenting questions about medical settings, we showed an explicit trigger warning (part 2 in **Fig S1B**). All participants chose to continue past this page.

"This questionnaire is about experiences in medical settings. The topic may trigger negative emotions if you have had negative experiences. You need to make sure that you have enough stability and support to handle emotions triggered by reading questions about sensory inputs and communication in medical settings. Because the questionnaire is anonymous, we have no preparedness or possibility to help you in

person. This means that you should not participate if there is a risk that you are triggered in a way that threatens your well-being. You may stop at any time by closing the browser window, and we would like you to do so straight away if you are not sure."

Branch questions

On this page (part 3, **Fig S1B**), we first asked the participants to indicate which of four settings they would like to see questions for. The settings included 1) somatic outpatient care, 2) psychiatric outpatient care, 3) somatic inpatient care, and 4) psychiatric inpatient care. This strategy allowed participants to skip settings that they had no experience of (e.g., if they never had been admitted to a hospital, they would not mark inpatient settings). This way, we avoided difficulties with interpreting missing data on the upcoming pages. On the same page we also asked participants to indicate whether 1) they had a diagnosis of autism, 2) someone suspected they were autistic, or 3) they were not autistic. The response to this question was used to hide autism-specific questions for non-autistic participants automatically. We needed these questions because the information provided in the demographic questionnaire could not automatically be carried over to the healthcare questionnaire.

Sensory questions

In multiple choice questions (described in the main article), we asked participants to what extend they agreed with a number of statements. **Table S1** shows a complete list of statements about sensory inputs in different room types and settings. Each participant saw only statements pertaining to the settings they had marked as relevant. The questions were divided into sections according to room type, such that multiple-choice questions for the room type in psychiatric and/or somatic settings were followed by a room-specific open-ended text question: "Based on your experiences, how could the environment in [room

type] be improved?". At the bottom of the page, the same question was also asked about "hospital wards" in general.

Table S1. Statements about sensory inputs.

Room type	Setting	Sensory question
In waiting rooms	...in psychiatric outpatient settings<br>...in somatic outpatient settings	...I have usually found the light levels acceptable.<br>...I have usually found the background sounds acceptable.<br>...I have rarely been bothered by visual impressions (e.g., colors, art, decorations).<br>...I have rarely been bothered by sensory impressions from other people.<br>...I have usually felt safe.
In examination rooms	...in psychiatric outpatient settings<br>...in somatic outpatient settings	...I have usually found the light levels acceptable.<br>...I have usually found the background sounds acceptable.<br>...I have rarely been bothered by visual impressions (e.g., colors, art, decorations).<br>...the volume of the healthcare provider's voice has usually been at a moderate level.
In patient rooms (where you sleep)	...in psychiatric inpatient settings<br>...in somatic inpatient settings	...I have usually found the light levels acceptable.<br>...I have usually found the background sounds acceptable.<br>...I have rarely been bothered by visual impressions (e.g., colors, art, decorations).<br>...I have rarely been bothered by sensory impressions from other people.<br>...I have rarely experienced problems with how blankets or sheets feel on the skin. [excluded due to low N value]<br>...I have usually felt safe.
In common rooms	...in psychiatric inpatient settings<br>...in somatic inpatient settings	...I have usually found the light levels acceptable.<br>...I have usually found the background sounds acceptable.<br>...I have rarely been bothered by visual impressions (e.g., colors, art, decorations).<br>...I have rarely been bothered by sensory impressions from other people.<br>...I have rarely been bothered by smells. [excluded due to low N value]<br>...I have usually felt safe.

Note. The expressions above were combined into statements presented to participants together with the multiple-choice options *Disagree, Somewhat Disagree, Neutral, Somewhat Agree, and Agree.*

Quality control

We considered any signs of inattention to be a reason for exclusion. To avoid subjective bias in exclusion of individuals from the study, the clinical information was not included during the first steps of quality control. Responses were first examined for completeness, and 6 participants were excluded because they failed to respond to all AQ and QoL items. Three participants were excluded because they provided unrealistic or inconsistent information (e.g., 170 children), even if the responses appeared to be typographical errors. When the clinical information was examined, 7 respondents were excluded because they disclosed a lack of knowledge about their own diagnoses, and another 5 were excluded based on providing conflicting or incomplete information about autism status.

Overall, we assumed that participants were honest and did not willingly sabotage the study. Nevertheless, we designed the questionnaire with the aim to minimize influence of inattentive participants. We did not include specific trap questions or explicit attention checks, because we have previously learned from similar autistic populations that such questions can be perceived as confusing or offensive. Instead, the length and complexity of the questionnaire likely filtered out participants with low motivation. This naturally caused a bias towards motivated subjects, but we preferred this over quality concerns, especially given the anonymous nature of the data.

Twenty-two respondents indicated that they or someone else suspected they were autistic, but that they did not have a formal diagnosis. Three of these had been denied referral for evaluation by the primary care system, three had been referred but not yet evaluated, and one had been evaluated without getting an autism diagnosis. The most endorsed reasons for not having a diagnosis were “I do not look autistic” and “I don’t know if I will be taken seriously by the healthcare system”. Because this group was heterogeneous and impossible to stratify further, we excluded the data from the current analysis.
